# Mendelian randomization analysis reveals genetic evidence for a causal link between inflammatory bowel disease and uterine cervical neoplasms

**DOI:** 10.3389/fgene.2024.1436512

**Published:** 2025-01-28

**Authors:** Chunge Cao, Xiaorui Sun, Xiaohu Chen, Ying Zhang, Chaoyan Yue

**Affiliations:** ^1^ Department of Obstetrics and Gynecology, Second Affiliated Hospital of Zhengzhou University, Zhengzhou, Henan, China; ^2^ Shanghai YK Pao School, Shanghai, China; ^3^ Obstetrics and Gynecology Hospital of Fudan University, Shanghai, China

**Keywords:** inflammatory bowel disease, Crohn’s disease, ulcerative colitis, uterine cervical neoplasm, Mendelian randomization

## Abstract

**Background:**

Inflammatory bowel disease (IBD) has been reported to be associated with risk of uterine cervical neoplasm. We aimed to evaluate the causal relationship between IBD and uterine cervical neoplasm using a bidirectional Mendelian randomization analysis.

**Methods:**

We derived instrumental variables for IBD, including Crohn’s disease and ulcerative colitis, from the IEU Open genome-wide association study (GWAS) database, and for the histological subtypes of uterine cervical neoplasm from the FinnGen repository’s GWAS data. The collected GWAS data predominantly represent individuals of European ancestry. The inverse-variance weighted (IVW) method was employed as primary analysis approach.

**Results:**

IBD (IVW odds ratio = 1.127, 95% confidence interval = 1.016–1.251; *p* = 0.024) and CD (IVW odds ratio = 1.119, 95% confidence interval = 1.023–1.224; *p* = 0.014) exhibited a significant causal effect on malignant cervical carcinoma. Sensitivity analyses confirmed these findings.

**Conclusion:**

Genetically predicted IBD and CD are risk factors for the development of malignant cervical carcinoma. Patients with IBD and CD require specific attention to prevent cervical squamous cell carcinoma. Further studies to elucidate the underlying mechanisms may reveal new therapeutic targets.

## Introduction

The primary subtypes of inflammatory bowel disease (IBD) are Crohn’s disease (CD) and ulcerative colitis (UC), both of which are immune-mediated chronic inflammations of the digestive tract that tend to recur throughout life (Kobayashi et al., 2020; Nadeem et al., 2020; Petagna et al., 2020). IBD has become a worldwide illness with a significant economic cost, with prevalence rates above 0.3% in several nations (Ng et al., 2017). Patients with IBD are much more likely to develop both intestinal and extraintestinal malignancies, particularly colorectal cancer and lymphomas, which are the cancers most frequently associated with IBD (Nadeem et al., 2020). According to a statewide study conducted in Finland, the mortality rates for malignant tumors in individuals with CD and UC were 24% and 23%, respectively (Jussila et al., 2014).

Cervical cancer ranks fourth globally in terms of both the number of diagnoses and deaths among female cancers (Bray et al., 2018). The primary cause of uterine cervical neoplasm is chronic infection with high-risk oncogenic subtypes of the human papillomavirus (HPV) (Muñoz et al., 2003). According to the World Health Organization, atypical cells in the uterine cervix caused by HPV infection typically take 15–20 years to develop into cervical cancer; this period shortens to 5–10 years in individuals with compromised immune systems (Thomas, 2023). HPV does not always lead to cervical cancer, nor is cervical cancer always caused by HPV. Given the high global burden of the disease, it is crucial to identify other risk factors for cervical cancer and those that can interact with HPV infection to prevent its onset and progression.

Numerous epidemiological studies have investigated the correlation between IBD and the risk of cervical cancer, but the results have been inconsistent. A case-controlled cohort study and meta-analysis have shown that, compared to normal controls, women with IBD have a higher chance of developing cervical abnormalities such as cancer and high-grade dysplasia (Goetgebuer et al., 2021; Kim et al., 2023b). Another meta-analysis study found a positive correlation between IBD and the risk of uterine cervix abnormalities; however, it did not find a significant correlation between IBD and the risk of cervical cancer (Cui et al., 2021). In South Korea, older patients (≥60 years) with newly diagnosed UC had a higher prevalence of cervical cancer (Kim et al., 2023a). However, another meta-analysis reveals that people with IBD do not have a statistically significant elevated risk of cervical cancer (Mann et al., 2022). In traditional observational studies, it is difficult to draw firm conclusions due to potential confounding and reverse causality. Therefore, the precise impact of IBD on the development of cervical carcinogenesis remains undetermined.

Mendelian randomization (MR) is a unique epidemiological technique used to determine the causal link between an exposure and an outcome of interest. In this approach, single nucleotide polymorphisms (SNPs) are used as instrumental variables (IVs). MR is less susceptible to reverse causation or confounding compared to conventional observational techniques (Smith and Ebrahim, 2003), as SNPs are randomly assigned to individuals via gamete development and conception and are unaffected by the onset or progression of the outcome (Smith and Hemani, 2014). In this study, we estimated the possible relationships between IBD and the risk of uterine cervical neoplasm and examined the direction of these relationships using a two-sample bidirectional MR design, aiming to provide scientific evidence for the prevention of uterine cervical neoplasm.

## Methods

### Study design

**FIGURE 1:**
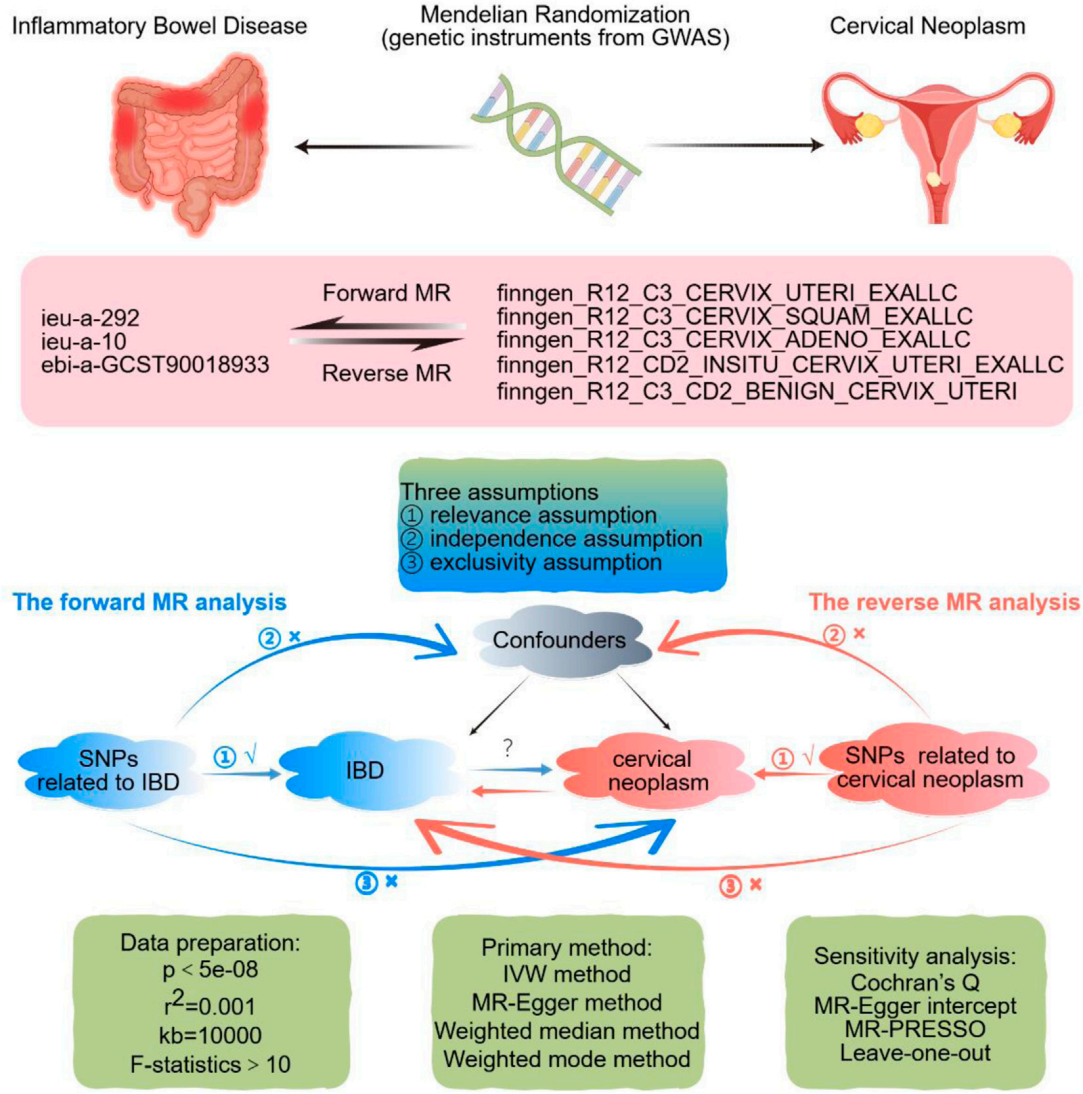
Design strategies for this Mendelian-randomization study between inflammatory bowel disease and uterine cervix neoplasm. The sign “√” suggests that SNPs are closely connected with the exposure. The sign “×” suggests that SNPs are disconnected from confounders or do not directly affect the outcome. Abbreviations: GWAS, genome-wide association study; IBD, inflammatory bowel disease; MR, Mendelian randomization; SNP, single nucleotide polymorphism; IVW, inverse-variance weighted. By FigDraw.

Figure 1 presents a brief description of design strategies for this bidirectional MR study between IBD and cervical neoplasm. This bidirectional two-sample MR study uses summary-level data from large-scale genome-wide association study (GWAS) studies. Exposure-related IVs were SNPs extracted from the GWAS data. The MR framework is based on three fundamental assumptions. The first assumption, relevance, is that there is a strong association between the IVs and the relevant exposure. The second assumption, independence, states that there is no unmeasured confounder that affects both the IVs and the outcome. Third, the assumption of exclusion restriction states that the IVs affect the outcome only through their effect on the exposure of interest (Lawlor et al., 2008).

### Data sources

The GWAS data for the IBD (GWAS ID: ieu-a-292), CD (GWAS ID: ieu-a-10), and UC (GWAS ID: ebi-a-GCST90018933) were obtained from the IEU open GWAS project. Five GWAS datasets of uterine cervical neoplasm histological subtypes, including malignant carcinoma, squamous cell carcinoma, adenocarcinoma, carcinoma *in situ*, and benign neoplasm, were downloaded from the FinnGen database (https://r12.finngen.fi/). Most of the participants in our data pool for MR analysis were of European ancestry. The details of the GWAS data used in this MR study are presented in Table 1.

**TABLE 1:** Information of summary-level genome-wide association studies used in this study.

Phenotypes	GWAS id/name	Ethnicity	Sample size	
			Cases	Controls
Inflammatory bowel disease	ieu-a-292	European	38,565	37,747
Crohn’s disease	ieu-a-10	European	14,763	15,977
Ulcerative colitis	ebi-a-GCST90018933	European	5,371	412,561
Cervical malignant carcinoma	Malignant neoplasm of cervix uteri (controls excluding all cancers)	European	878	222,078
Cervical squamous cell carcinoma	Squamous cell neoplasms and carcinoma of cervix (controls excluding all cancers)	European	222	222,078
Cervical adenocarcinoma	Adenocarcinomas of cervix (controls excluding all cancers)	European	142	222,078
Carcinoma *in situ* of cervix	Carcinoma *in situ* of cervix uteri (controls excluding all cancers)	European	3,701	219,058
Cervical benign neoplasm	Other benign neoplasm of uterus: Cervix uteri	European	207	281,857

We selected SNPs that were intensely associated with exposure at a genome-wide significance threshold of P < 5e-08. We used the European ancestry data from the 1000 Genomes Project (RRID: SCR_008801) as our reference and used strict clumping settings with kb > 10,000 and *r* ^2^ < 0.001 to reduce linkage disequilibrium among variables. In order to ensure the precision of the findings, we removed palindromic SNPs with intermediate allele frequencies. By searching the human genotype-phenotype association database PhenoScanner V2, SNPs linked to possible confounding variables of the outcomes, such as diabetes mellitus, alcohol use, smoking, and obesity, were located and excluded. We eliminated the weak instrumental variables using the F statistic in order to guarantee a strong correlation between exposure factors and IVs. The *F* statistic was calculated as the square of the beta divided by the variance for the SNP-exposure association, and a value of *F* > 10 was considered to satisfy the criteria for a strong association (Bowden et al., 2016).

### Statistical analysis

We used four methods to evaluate the causal relationship between IBD and uterine cervical neoplasm: inverse-variance weighted (IVW), MR-Egger, weighted median, and weighted mode. If these methods produce inconsistent results, we prioritize IVW as the primary result, but we only consider the IVW results to be reliable if the results of other methods are directionally similar to the IVW result. The MR-Egger intercept test was performed to identify directional pleiotropy (Bowden et al., 2015). The MR pleiotropy residual sum and outlier test (MR-PRESSO) method was utilized to evaluate and correct horizontal pleiotropy as well as find the outlying SNPs. The Cochran’s Q test was used to assess heterogeneity among the selected SNPs. The leave-one-out sensitivity analysis was used to investigate whether a single SNP contributed to bias and affected the overall causal effect (Hu et al., 2023). All the data analysis was performed using TwoSampleMR (v.4.3.1), a *R* statistical software program that implements the two-sample MR technique. Causal estimates are shown using odds ratios (ORs) and 95% confidence intervals (CI). A two-tailed *p*-value less than 0.05 was used to establish a statistically significant difference.

## Results

### Genetic instruments and strength

**FIGURE 2:**
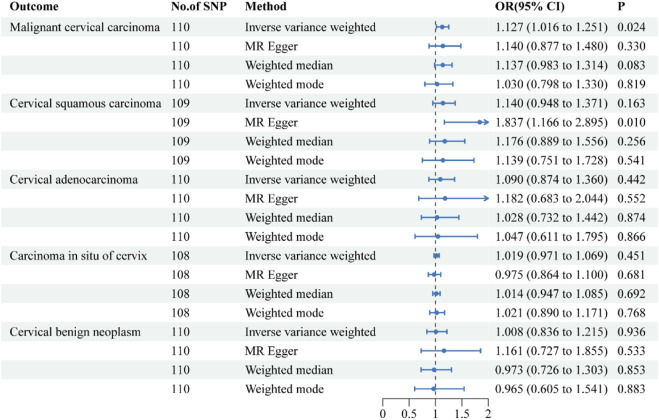
Forest plots of the causal effect of inflammatory bowel disease on the risk of uterine cervical neoplasm. The results are shown for the different Mendelian randomization analysis methods used in this study. Abbreviations: No., number; IVW, inverse-variance weighted; OR, odds ratio; CI, confidence interval.

**FIGURE 3:**
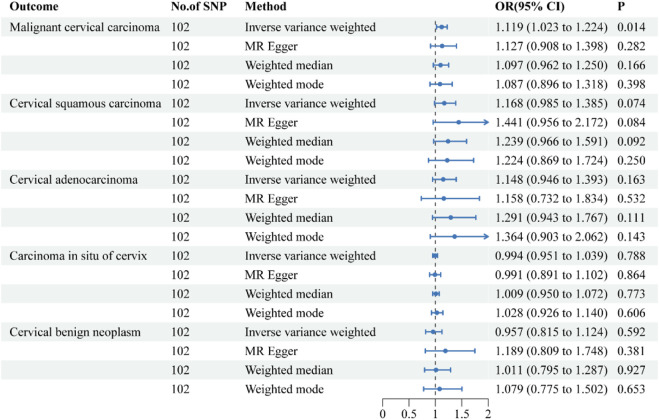
Forest plots of the causal effect of Crohn’s disease on the risk of uterine cervical neoplasm. The results are shown for the different Mendelian randomization analysis methods used in this study. Abbreviations: No., number; IVW, inverse-variance weighted; OR, odds ratio; CI, confidence interval.

**FIGURE 4:**
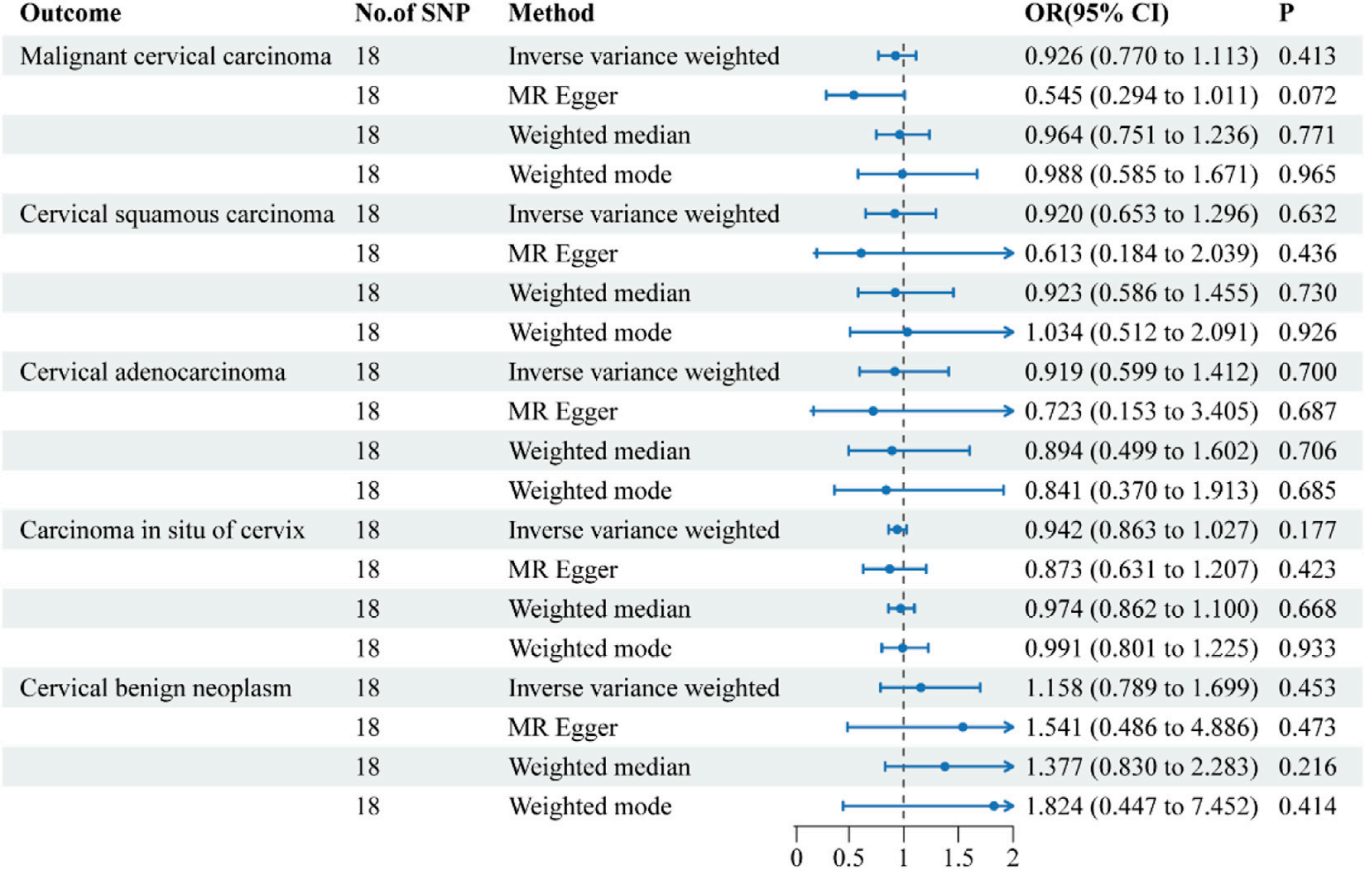
Forest plots of the causal effect of ulcerative colitis on the risk of uterine cervical neoplasm. The results are shown for the different Mendelian randomization analysis methods used in this study. Abbreviations: No., number; IVW, inverse-variance weighted; OR, odds ratio; CI, confidence interval.

In the forward MR analysis, SNPs associated with IBD, CD, and UC are presented in Figures 2–4. The visualized data plots in this study, including scatter plots, forest plots, leave-one-out analyses, and funnel plots, are presented in Supplementary Figures S1, S2. Detailed information about these SNPs is listed in Supplementary Tables S1–S5. In the reverse MR analysis, we relaxed the genome-wide significance threshold for the SNPs associated with uterine cervical neoplasm to a maximum of 5e-05. However, after linkage disequilibrium clumping and data harmonization, there were not enough SNPs available for estimating the causal effect of uterine cervical neoplasm on IBD. Therefore, we did not perform the MR analysis to assess the causality of cervical cancer on IBD. The *F* statistics for each selected SNP exceeded 10, suggesting that the weak instrument bias was not statistically significant.

### The causal relationship between IBD and uterine cervix neoplasm

The causal effects of IBD on the risk of uterine cervical neoplasm are illustrated in Figure 2. The findings of the sensitivity analysis are shown in Table 2. The results of the IVW method indicated that a 1-logOR increase in IBD significantly increased the risk of malignant cervical carcinoma (OR = 1.127, 95% CI = 1.016–1.251; *p* = 0.024). The MR-Egger and weighted median methods showed similar directional associations between IBD and malignant cervical carcinoma, although these results were not statistically significant. Cochran’s Q test indicated the presence of heterogeneity in the individual effect estimates generated by each SNP (*p* = 0.008), but the random-effects IVW model was not affected. The MR-Egger intercept test did not suggest the presence of directional pleiotropy (*p* = 0.930). The MR-PRESSO test showed no outliers. The robustness of our results was confirmed by the leave-one-out sensitivity test.

**TABLE 2:** Sensitivity analysis of the causal effect of inflammatory bowel disease on uterine cervical neoplasm.

Outcome	IBD		CD		UC	
	*p*_Cochran’s Q_	*p*_MR-Egger_	*p*_Cochran’s Q_	*p*_MR-Egger_	*p*_Cochran’s Q_	*p*_MR-Egger_
Malignant cervical carcinoma	0.008	0.930	0.020	0.943	0.306	0.099
Cervical squamous cell carcinoma	0.278	0.027	0.080	0.273	0.942	0.499
Cervical adenocarcinoma	0.655	0.754	0.529	0.032	0.865	0.756
Carcinoma *in situ* of cervix	0.106	0.438	0.018	0.947	0.394	0.640
Cervical benign neoplasm	0.372	0.519	0.967	0.227	0.232	0.613

Abbreviations: IBD, inflammatory bowel disease; CD, Crohn’s disease; UC, ulcerative disease.

The IVW method suggested a trend towards a causal relationship between IBD and cervical squamous cell carcinoma (OR = 1.140, 95% CI = 0.948–1.371), although this was not statistically significant (*p* = 0.163). In contrast, the MR-Egger method demonstrated a clear and significant causal relationship (OR = 1.837, 95% CI = 1.166–2.895, *p* = 0.01), but the intercept test indicated the presence of directional pleiotropy (*p* = 0.027).

The IVW results revealed that IBD had no causal effects on cervical adenocarcinoma, carcinoma *in situ* of cervix, or cervical benign neoplasm. The results of the other three methods were consistent with the IVW findings. Sensitivity analysis confirmed the reliability of our results.

### The causal relationship between CD and uterine cervix neoplasm

The causative effect estimates of CD on the risk of uterine cervical neoplasm are presented in Figure 3. The sensitivity analysis results are shown in Table 2. The IVW results suggested a significant causal effect of CD on the risk of malignant cervical carcinoma (OR = 1.119, 95% CI = 1.023–1.224; *P* = 0.014). The results of the MR-Egger, weighted median, and weighted mode methods showed directionally similar results without statistical significance. Cochran’s Q test indicated the presence of heterogeneity in the individual effect estimates generated by each SNP (*p* = 0.020). The directional pleiotropy was not supported by the MR-Egger intercept test (*P* = 0.273). The MR-PRESSO tests detected no outliers. The leave-one-out sensitivity test validated the robustness of these findings.

All four methods suggested a trend towards a causal effect of IBD on cervical squamous cell carcinoma and cervical adenocarcinoma, although it was not statistically significant. The IVW results revealed that CD had no causal effects on carcinoma *in situ* of cervix, or cervical benign neoplasm.

### The causal relationship between UC and uterine cervix neoplasm

The causative effect estimates of UC on the risk of uterine cervical neoplasm are presented in Figure 4. The sensitivity analysis results are shown in Table 2. The MR analysis revealed no causal effect of UC on any histological subtype of uterine cervical neoplasm. The MR-Egger intercept tests detected no directional pleiotropy. The Cochran’s Q tests observed no heterogeneity in the individual effect estimates generated by each SNP. The MR-PRESSO tests provided no indications of horizontal pleiotropy or outliers. The leave-one-out sensitivity tests confirmed the robustness of these results.

## Discussion

This study was the first to extensively examine the causal relationship between IBD (including UC and CD) and uterine cervical neoplasia using summary-level GWAS data. MR results revealed that IBD and CD have a strong causal effect on the incidence of malignant cervical cancer, whereas UC had no influence.

The findings of this study were consistent with those of several previous observational studies on the relationship between IBD and cervical cancer risk. A nationwide matched-cohort study between 1979 and 2011 that included 8,717 women diagnosed with CD, 18,691 with UC, and 1,508,334 healthy controls from the general population showed that CD patients had increased incidence rate ratios (IRRs) for cervical cancer (IRR = 1.53; 95% CI = 1.04–2.27), whereas UC patients did not (Rungoe et al., 2015). Similarly, a study on cancer risk in a well-defined IBD cohort from the population of North Jutland County, Denmark, indicated a higher standardized incidence ratio (SIR) for cervical dysplasia (including carcinoma *in situ*) in patients with CD (SIR = 1.65; 95% CI = 1.10–2.37) but not in those with UC (SIR = 0.71; 95% CI = 0.43–1.11) (Jess et al., 2013). Moreover, a record linkage study to determine the risk of cancers in IBD cohorts revealed an increased rate ratio (RR) for cervical cancer in patients with CD (RR = 2.63; 95% CI = 1.12–5.29) but not UC (RR = 1.91; 95% CI = 0.69–4.24) (Goldacre et al., 2008). However, studies also showed that IBD did not increase the risk of uterine cervical cancer (Cui et al., 2021). Variations in analytical techniques, sample sizes, genetic backgrounds, cohort composition, and other factors might be blamed for the disparate outcomes of observational research. Considering that observational studies were unable to confirm the causal relationship between exposure and outcome and might be susceptible to confounding factors, we conducted this MR study to evaluate the causality between IBD and cervical cancer. This MR study could eliminate some of the common sources of bias, such as confounding, reverse causation, and measurement error, and achieve reliable causal estimation based on the three core IV assumptions.

A previous MR study conducted by Gao et al. on the causal association between IBD and 32 site-specific extracolonic cancers found no evidence of causality between IBD (including CD and UC) and uterine cervical cancer (Gao et al., 2023). The following considerations may partly account for the discrepancies between that study and ours: First, the GWAS datasets for IBD used for analysis in the two studies were different, although they were both released by the International IBD Genetics Consortium. Second, the GWAS datasets for cervical cancer used for analysis in the two studies were also different. In our study, the GWAS data for cervical cancer were derived from the FinnGen R12 database and were histologically classified for cervical cancer. However, the GWAS data for cervical cancer in the Gao et al. study came from the UK Biobank and the FinnGen R8 database, and cervical cancer was not histologically classified. Third, the inclusion criteria for IVs were different. Although both studies set the genome-wide significance level of P < 5e-08, we used the criteria of clump windows >10,000 kb and r^2^ < 0.001 to eliminate linkage disequilibrium. In contrast, the criteria used by Gao et al. were clump windows >5,000 kb and r^2^ < 0.01. In this study, we used four different MR analysis methods to determine the causal relationship between IBD and cervical cancer risk, which enhanced the reliability of our results. The sensitivity analysis by Cochran’s Q test, the MR-Egger intercept test, the MR-PRESSO test jointly confirmed the robustness of our results.

There are several potential mechanisms that may explain the causal association between IBD and malignant cervical cancer. Firstly, the close anatomical proximity may facilitate the spread of inflammation from the gut to the cervix. Chronic inflammation might be a possible risk factor for cervical dysplasia progressing to cervical cancer (Afsar et al., 2023). CD is a chronic inflammatory condition that frequently involves the deeper layers of the entire intestine, particularly the distal ileum (Petagna et al., 2020). On the other hand, ulcerative colitis is characterized by inflammation and ulcers in the lining of the large colon and the rectum (Kobayashi et al., 2020). We found that CD patients had a higher risk of malignant cervical cancer than UC patients, which could be due to the more extensive inflammation in CD patients and their more frequent use of immunosuppressive agents compared with UC patients.

Secondly, immunosuppressive medication may partly account for the causal relationship between IBD and cervical cancer. Immunosuppressive drugs have been demonstrated to have chromosomal breakage, immune response depression, mutagenic, teratogenic, and even carcinogenic effects in experimental animals (Reyes et al., 2023). Previous studies have shown that immunosuppressive therapy for IBD may increase the cancer risk (Beaugerie and Kirchgesner, 2019). Moreover, IBD patients who received immunosuppressive medication had a higher chance of developing medium-high-risk cervical abnormalities or cancer than those who did not (Allegretti et al., 2015). The risk of cervical cancer may be higher for IBD patients who use immunosuppressive drugs, but the absolute risks of these complications are still low, and the decision to discontinue treatment should be carefully weighed against the increased risks of other complications due to undertreated IBD (Kaplan et al., 2023). The European Crohn’s and Colitis Organization advises that female IBD patients who take immunosuppressive agents should get annual cervical cancer screening, and HPV vaccination is recommended routinely for both young female and male IBD patients (Kucharzik et al., 2021).

Thirdly, IBD may increase cervical cancer risk by affecting the estrogen-mediated gut-vagina axis (Chang et al., 2023). The gut microbiota, which is altered in IBD, influences the metabolism of estrogen and the composition of the vaginal microbiota, both of which can modulate the tumor microenvironment and promote HPV-positive cervical cancer (Qiu et al., 2022). Cervical cancer is primarily attributable to persistent infection with high-risk oncogenic HPV types (Ibeanu, 2011). Certain histological forms of cervical malignancies are more susceptible to specific HPV types than others. Squamous cell carcinoma is most strongly correlated with HPV16, whereas adenocarcinoma is most correlated with HPV18 (Berrington De González et al., 2004). Nearly all clinically noticeable benign lesions, including genital warts and laryngeal papilloma, are caused by low-risk HPV types, particularly types 6 and 11 (Sanjosé et al., 2007). It can be hypothesized that different types of HPV may have different levels of response to inflammation, immunosuppressive agents, and vaginal microbiota, resulting in a stronger causal effect of CD on cervical squamous cell carcinoma than on other histological subtypes of cervical neoplasm.

Besides, oxidative stress is an important factor in the pathogenesis of IBD and cervical cancer. In IBD, oxidative stress is triggered by inflammatory responses, leading to the excessive production of reactive oxygen species (ROS). These ROS not only damage the intestinal mucosal barrier but also activate inflammatory mediators, further exacerbating inflammation and tissue damage. Similarly, in the development of cervical cancer, oxidative stress promotes DNA damage, genomic instability, and cell proliferation, creating favorable conditions for tumor initiation and progression (Sahoo et al., 2023).

Our study has several notable strengths. First, this is the first study to use MR design to assess the causal relationship between IBD and its subtypes and the histological subtypes of cervical neoplasm. MR evidence bridges experimental and observational studies, which could provide more robust evidence for the causality between exposure and outcome. Second, we rigorously chose IVs that satisfied the core MR assumptions and avoided any possible weak instrument bias. To further guarantee the accuracy of our findings and to identify and correct any errors brought on by directional or horizontal pleiotropy, we also employed sensitivity analysis techniques like MR-PRESSO and MR-Egger. Third, in order to exclude any potential confounding from a more diversified population, we limited the genetic origin of the individuals to mostly European ancestry.

Although our study highlights the significant contribution of CD to the development of cervical squamous cell carcinoma, the practical implications of these findings for public health and clinical practice need to be addressed. To better translate these findings into actionable steps, we recommend: enhanced education and awareness through medical institutions and community health centers to raise awareness among CD patients about the importance of HPV vaccination and cervical cancer screening; specific screening guidelines for CD patients, including the frequency, methods, and follow-up measures; optimized healthcare processes to ensure that CD patients have easy access to HPV vaccination and cervical cancer screening services, such as setting up dedicated screening and consultation sessions in gastroenterology clinics; and policy support to advocate for policies that provide financial support and insurance coverage for HPV vaccination and cervical cancer screening for CD patients. Through these measures, we can better translate our research findings into practical public health interventions, effectively reducing the risk of cervical cancer in CD patients.

Nevertheless, we would like to acknowledge some limitations. First, the publicly accessible GWAS data lacked the unique characteristics of IBD, such as the severity, prognosis, and course of therapy. Therefore, we were unable to perform a stratified MR analysis with the available information. Additionally, the integration of clinical variables, such as the use of immunosuppressive medications and HPV infection rates, would enhance the interpretability of our findings (Kucharzik et al., 2021). We plan to explore the collaboration between genetic findings and clinical data in future studies, particularly focusing on the impact of HPV infection, to gain a deeper understanding of the underlying mechanisms. Second, the GWAS datasets utilized in this study predominantly consist of individuals of European descent, which constrains the generalizability of the findings to other populations. This limitation is especially pertinent in regions with a higher prevalence of cervical cancer and distinct genetic backgrounds. Consequently, additional research is necessary to validate the applicability of our findings across a broader demographic spectrum. Third, we could not completely eliminate the influence of horizontal pleiotropy, despite using several stringent methods to identify and avoid outlier SNPs that induce horizontal pleiotropy. This may be attributed to the complex biological functions of many SNPs. Fourth, while MR study could imply the causal effects of IBD and CD on malignant cervical carcinoma, the underlying mechanisms remain to be elucidated. Future studies should aim to elucidate the precise molecular and cellular interactions that underlie this association, providing a more comprehensive understanding of the pathogenesis of malignant cervical carcinoma in the context of IBD, CD and other immunosuppressive conditions. Fifth, some of the analyses in this study used a relatively small sample size, with only a few hundred participants. This small sample size may reduce the confidence in our conclusions. Although we utilized the Finngen R12 dataset, which is the largest available sample for this specific condition, the sample size remains limited. If larger datasets become available in the future, we will further validate and strengthen our findings. Sixth, this study primarily discusses the relationship between IBD and cervical neoplasms at the phenotypic level, more in-depth Mendelian randomization analyses, such as exploring the mediating role of immune cells, would help further elucidate the biological mechanisms underlying this association. In future research, we will consider conducting these complex analyses to provide a more comprehensive understanding.

## Conclusion

In conclusion, our study showed that IBD and CD significantly contributed to the development of cervical squamous cell carcinoma. All IBD and CD women should be strongly encouraged to receive HPV vaccinations and regular cervical cancer screenings in order to reduce their risk of developing cervical cancer. Especially for IBD and CD women with HPV infection, the frequency of cervical cancer screening should be increased.

## Data Availability

The original contributions presented in the study are included in the article/Supplementary Material, further inquiries can be directed to the corresponding authors.
