# Supplementary material for: Mendelian randomization analysis reveals genetic evidence for a causal link between inflammatory bowel disease and uterine cervical neoplasms
Source: Front Genet. 2025 Jan 28;15:1436512. doi: 10.3389/fgene.2024.1436512 (PMC11810950; doi:10.3389/fgene.2024.1436512)
Supplement: Supplementary file 1 [file Table2.docx]

**
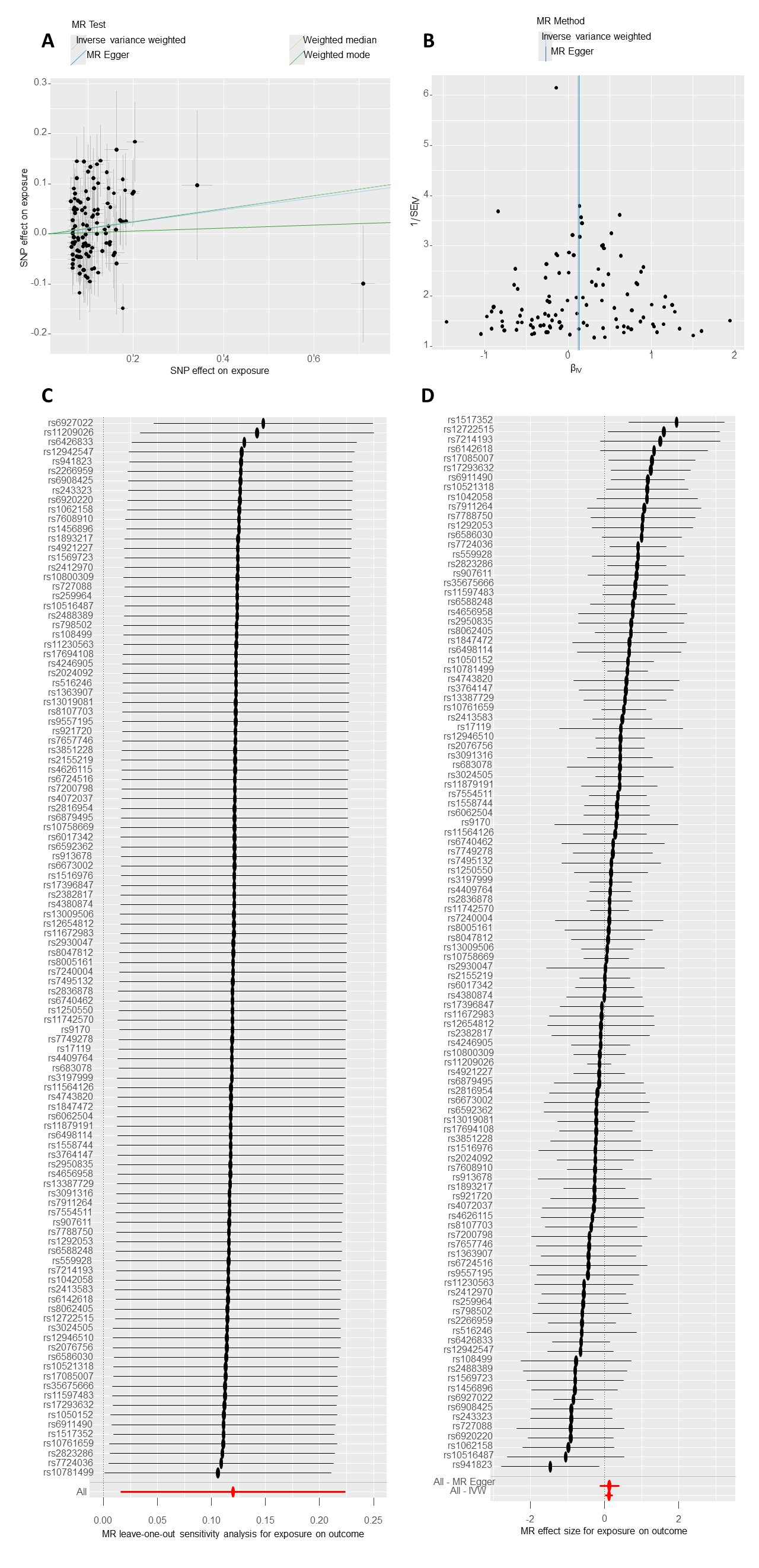
**

**Figure S1**. Casual effect of IBD on Cervical malignant carcinoma. (A) Scatter plot of the association between IBD and cervical malignant carcinoma. (B) Funnel plot to show the causal effect size estimate of IBD on Cervical malignant carcinoma (red line segment) and 95% CI values (gray line segment) for each SNP. (C) Leave-one-out analyses to evaluate the impact of each SNP on the overall result. (D) Forest plot to detect obvious heterogeneity and system bias. IBD, inflammatory bowel disease; IVW, inverse variance weighted; SNPs, single-nucleotide polymorphisms.


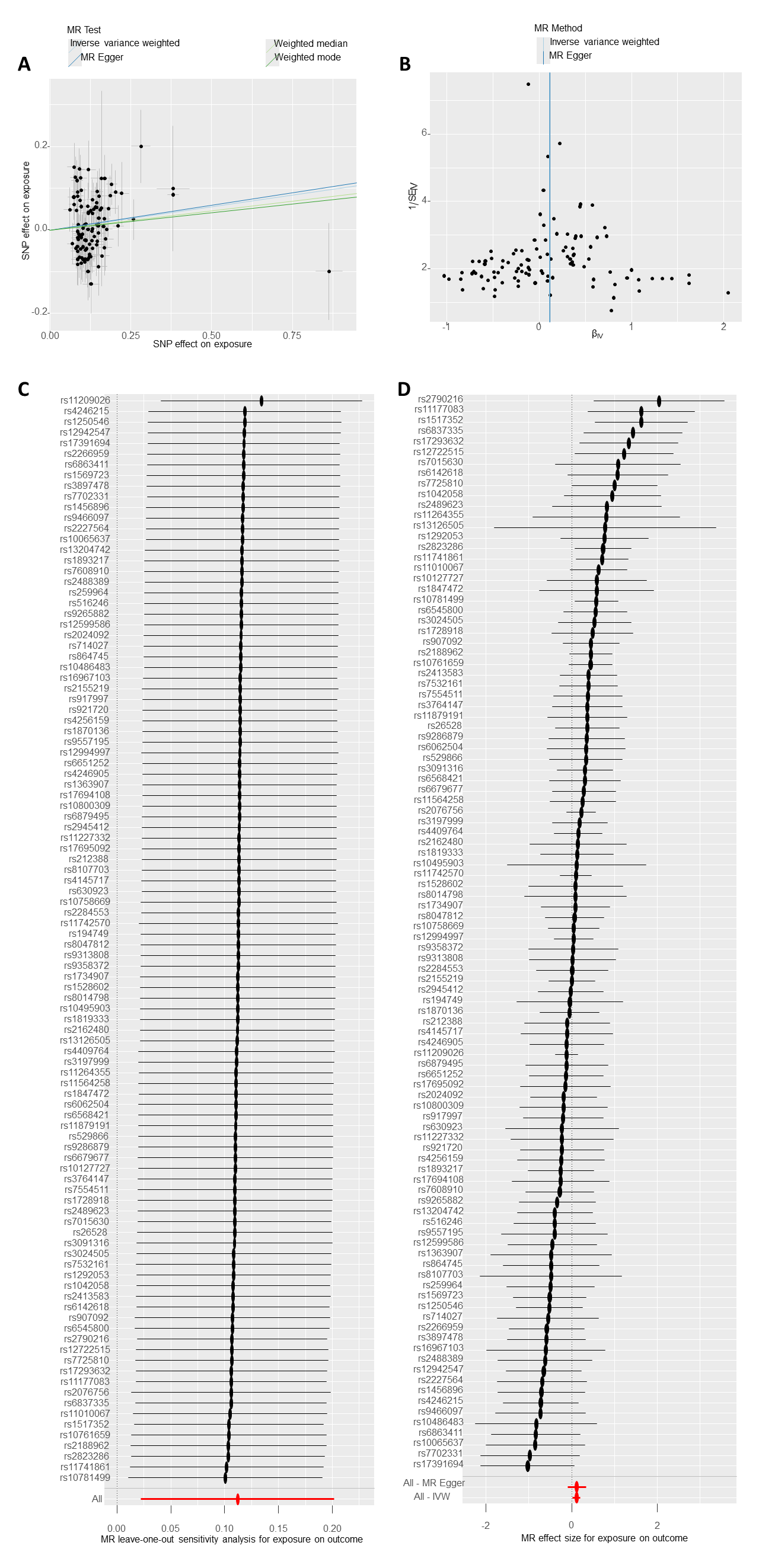


**Figure S2.** Scatter plot (A), funnel plot (B), sensitivity analysis (C) and (D) forest plot of the causal effect of IBD on risk of cervical squamous cell carcinoma.
